# Temporal modulation transfer functions in cochlear implantees using a method that limits overall loudness cues

**DOI:** 10.1016/j.heares.2011.11.009

**Published:** 2012-01

**Authors:** Matthew Fraser, Colette M. McKay

**Affiliations:** School of Psychological Sciences, The University of Manchester, Manchester M13 9PL, UK

**Keywords:** CI, cochlear implant, CL, current level, DL, difference limen, DR, dynamic range, *m*, modulation index, MCL, maximum comfortable level, MDT, modulation detection threshold, MP, monopolar, NIFC, n-interval forced choice, TMTF, temporal modulation transfer function

## Abstract

Temporal modulation transfer functions (TMTFs) were measured for six users of cochlear implants, using different carrier rates and levels. Unlike most previous studies investigating modulation detection, the experimental design limited potential effects of overall loudness cues. Psychometric functions (percent correct discrimination of modulated from unmodulated stimuli versus modulation depth) were obtained. For each modulation depth, each modulated stimulus was loudness balanced to the unmodulated reference stimulus, and level jitter was applied in the discrimination task. The loudness-balance data showed that the modulated stimuli were louder than the unmodulated reference stimuli with the same average current, thus confirming the need to limit loudness cues when measuring modulation detection. TMTFs measured in this way had a low-pass characteristic, with a cut-off frequency (at comfortably loud levels) similar to that for normal-hearing listeners. A reduction in level caused degradation in modulation detection efficiency and a lower-cut-off frequency (i.e. poorer temporal resolution). An increase in carrier rate also led to a degradation in modulation detection efficiency, but only at lower levels or higher modulation frequencies. When detection thresholds were expressed as a proportion of dynamic range, there was no effect of carrier rate for the lowest modulation frequency (50 Hz) at either level.

## Highlights

► Loudness cues lead to overestimation of modulation detection ability in cochlear implantees. ► Implantees show similar temporal resolution ability to normal-hearing listeners. ► Modulation sensitivity is poorer at higher carrier rates for some conditions. ► Modulation sensitivity is poorer at lower levels.

## 1 Introduction

The perception of amplitude envelope cues is important for the speech understanding of cochlear implant (CI) users. Several studies have been published in which modulation detection thresholds (MDTs) were measured and discussed in relation to signal processing or speech understanding (Busby et al., 1993; Cazals et al., 1994; Chatterjee and Oba, 2005; Chatterjee and Oberzut, 2011; Fu, 2002; Galvin and Fu, 2005, 2009; Pfingst et al., 2007, 2008; Shannon, 1992). However, the effect of modulation on overall loudness has implications for the interpretation of the results of these experiments. McKay and Henshall (2010) have shown that modulated pulsatile stimuli are louder than an unmodulated stimulus of equal average current. The potential effect of overall loudness cues on modulation detection can therefore bring into question inferences that have been made regarding, for example, the effect of carrier rate, carrier level, or modulation frequency on MDTs. In this paper, a modulation detection method that limited the use of overall loudness cues was used to re-investigate the effect of stimulus level, carrier rate and modulation frequency on modulation detection.

Previous studies that have measured MDTs for CI users have used sinusoidal current- or phase-duration-modulated stimuli in an adaptive n-interval forced-choice (NIFC) task. The mean currents of the modulated stimuli were equal to the current of the unmodulated stimulus. In most cases, the modulated stimuli were assumed to be of equal loudness to the unmodulated stimulus, and no procedures were applied to minimize overall loudness cues. That is, it was assumed that detection was achieved by detection of the modulation itself, without the use of alternative cues such as an overall loudness difference between modulated and unmodulated stimuli. Pfingst et al. (2007) did report that the modulated stimuli were louder than the unmodulated stimulus (as indicated by the lower average comfortable-loud levels), but they dismissed the possibility of overall loudness cues being utilized by subjects, with the argument that the variability in setting comfort levels by adjustment was greater than the average expected adjustment for equal loudness at the MDT, implying that any loudness change at MDT would not be detectable. However, the fact that the current differences at MDT may be smaller than the range of currents obtained in an adjustment task for comfortable level does not guarantee that the loudness difference is not detectable at MDT in the NIFC task; the smallest current level difference detectable in an NIFC discrimination task is far smaller than the variability in comfort levels. More recently, Galvin and Fu (2009) noted in their discussion that there might be overall loudness cues in amplitude modulation detection. They did not, however, actively test this possibility or take any action to minimize potential loudness cues when collecting their data. Chatterjee and Oberzut (2011), using a carrier rate of 2000 pps and modulation frequencies between 20 and 200 Hz, demonstrated that the use of level jitter can result in poorer MDTs.

McKay and Henshall (2010) demonstrated both with a model and experimental data that the effect of amplitude modulation on the loudness of a pulse train differs with the absolute current levels used. The results showed that, for low currents (stimuli with carrier rates of 8000 Hz and above at all levels in the dynamic range, or lower-rate stimuli near threshold), modulated stimuli with average currents equal to that of the reference unmodulated stimulus had loudness approximately equal to that of the reference. However, for higher absolute currents (stimuli above threshold with rates below 8000 Hz), the modulated stimulus was louder than the unmodulated one. Overall loudness increased with modulation depth (keeping average current constant), and the size of this effect depended on both level within the dynamic range (DR) and carrier rate, increasing with higher levels (keeping carrier rate constant) and with lower carrier rates (keeping level in the DR constant). Thus if modulation detection itself was unaffected by carrier rate or level, and if overall loudness cues were detectable by the subjects, then it would be expected that they would perform the discrimination task better at higher levels and lower carrier rates. This pattern of results has indeed been found: higher levels are associated with better performance (Chatterjee and Robert, 2001; Chatterjee and Oberzut, 2011; Fu, 2002; Galvin and Fu, 2005, 2009; Pfingst et al., 2007); and lower carrier rates are associated with better performance (Galvin and Fu, 2005, 2009; Pfingst et al., 2007). Therefore the potential role of overall loudness cues in these studies brings some uncertainty to their interpretation.

The discrimination task itself allows the use of any available cue or combination of cues to select the different stimulus. Any overall loudness cue should be limited if the MDTs are to have meaning for information transmission in ecologically relevant situations such as listening to speech, where overall levels vary dynamically. The addition of the overall loudness cue will lead to improved MDTs since it co-varies with modulation depth. The relative contribution of modulation or loudness cues to detection thresholds when loudness cues are not limited will depend on the relative salience of the cues in particular conditions. Since factors that can influence modulation detection ability (level, carrier rate, and modulation frequency) may also affect the salience of the loudness cue, and will differ between subjects, it is difficult to precisely predict for any one case what the effect of loudness cues would be on the modulation-detection task.

There are only two predictions that can be made with any degree of certainty: that loudness cues, when present and salient, will lead to overestimation of modulation detection ability; and that this overestimation is very likely to influence measurements at high modulation frequencies. At high modulation frequencies, for which modulation detection would be expected to be very poor, and loudness cues uninfluenced, compared to lower modulation frequencies, loudness cues might dominate or even be the sole cue. It would then be expected that MDTs would have a minimum value (as modulation frequency is increased) that is governed by the loudness cue for the given carrier rate and level. Thus the temporal modulation transfer functions (TMTFs, the functions relating MDTs to modulation frequency) would be predicted to have a low-pass characteristic for low modulation frequencies but to flatten out to a constant value at the modulation frequency at which loudness cues became dominant over modulation cues. This prediction is subject to the effect of modulation on loudness being independent of modulation frequency for high modulation frequencies, as predicted by the model and data of McKay and Henshall (2010). Cazals et al. (1994) measured TMTFs using modulation frequencies between 20 and 800 Hz at three different current levels and the functions did have the characteristics predicted above: subjects showed no decrease in modulation sensitivity between 200 and 800 Hz modulation. The detection thresholds for high modulation frequencies corresponded very well to those expected if subjects were exclusively using overall loudness cues (McKay and Henshall, 2010). It was hypothesized in the current experiment, where loudness cues were limited, that all subjects would exhibit a TMTF with a low-pass characteristic, even in conditions (such as low levels) for which modulation detection is poor.

The experiments reported here examined the effect of carrier rate, carrier level, and modulation frequency on modulation detection, while limiting overall loudness cues. The loudness cues were limited by first loudness balancing all modulated stimuli to the reference unmodulated stimulus, and then using an amount of level jitter that would limit the use of any residual loudness differences after loudness balancing.

## 2 Methods

### 2.1 Participants

Six implantees with the CI24RE (Freedom) implant manufactured by Cochlear Ltd. participated in the experiment. The details of their etiology and implant use are contained in Table 1. All were postlingually deaf adults, who had no useful hearing in the implanted ear before implantation. The table includes information about speech perception performance for these subjects.

### 2.2 Equipment and stimuli

Psychophysical procedures were performed and responses recorded using ImpResS software. The computer that controlled the experiment was interfaced with the implant via a Spear research processor. The stimuli consisted of trains of biphasic current pulses, each 500 ms in duration. Each pulse had a phase duration of 26 μs and an interphase gap of 8.4 μs. The mode of stimulation (electrode configuration) was always monopolar (MP1 + 2), and the active electrode was always electrode 14. The current values and current adjustments are reported in clinical current-level units (CL). For this implant (Freedom), each current level step represents a 0.157 dB change in current. Carrier rates were 600 and 2400 pps, and modulation frequencies (50, 150, and 300 Hz for the 600 pps stimuli, and 50, 150, 200, 300, 400, 480 and 600 Hz for the 2400 pps stimuli) were always sub-multiples of the carrier rates to avoid additional modulation due to aliasing and incomplete amplitude range sampling (McKay et al., 1994). Sinusoidal amplitude modulation on the current parameter was used. The sinusoidal waveform was sampled by the carrier pulses, with the first pulse placed at the waveform peak to ensure full sampling of the modulation depth (for example, the 300 Hz modulation in the 600 pps carrier had pulses that alternated between peak and trough amplitudes). Note that previous studies comparing carrier rates used 250 Hz as the lowest carrier rate (Pfingst et al., 2007; Galvin and Fu, 2005, 2009). However, to obtain TMTFs, modulation frequencies up to at least 300 Hz are necessary, so 600 pps was chosen as the lower carrier rate. Modulation depths (peak to trough) ranged from 1 to 50 CLs (−41 to −3 dB in 20log[m] units). The modulation index, *m*, denotes the amount of modulation on the current parameter, where *m* = 1 represents 100% modulation.

When adjusting the overall level of the modulated stimuli, all pulses in the pulse train were adjusted by an equal number of CLs (maintaining constant peak-to-peak modulation depth both in terms of current steps and as a proportion of the mean current).

### 2.3 Procedures

#### 2.3.1 Overview

TMTFs were determined at two current levels (40–50% and 80–100% of the DR) for each carrier rate. The MDT was defined as the modulation depth that was correctly detected 70% of the time using a method of constant stimuli and a three-interval, three-alternative, forced-choice task. To limit the use of loudness cues, all modulated stimuli (at each modulation depth) were first loudness balanced with the reference (unmodulated) stimulus. In addition, random level jitter was applied to all intervals in the modulation-detection task to prevent the use of residual loudness inequalities after balancing.

#### 2.3.2 Setting the reference levels

The levels of the unmodulated reference stimuli (see Table 2) were set for the four TMTFs (two carrier rates at two levels in the DR) as follows. The unmodulated reference stimulus at 600 pps carrier rate was first set to 80–100% of the DR. The maximum comfortable level (MCL) was obtained by presenting the reference stimulus in an ascending sequence. Subjects were instructed to indicate on a loudness category scale at what point the stimulus was ‘too loud’. The MCL was taken to be the highest level categorized at the next lower category (‘loud but comfortable’). The detection threshold of the same stimulus was measured using an adaptive three-interval, three-alternative, forced-choice procedure. Subjects were instructed to select which of three intervals contained a stimulus. The stimulus occurred in one randomly chosen interval. The current level of this stimulus was adjusted in a two-down/one-up procedure with steps of 4 CLs until the first two reversals, and 2 CLs for the remaining eight reversals. The threshold was taken as the average CL at the last six reversals. The task was repeated and the average of the two threshold estimates was calculated. The subject's DR was calculated in current steps (MCL minus threshold). The higher reference level for TMTF testing was set in the region 80–100%DR, by asking the subject to nominate the highest level that would be comfortable to complete the whole experiment, and the lower level (40–50%DR) was set by halving the sensation level (in CL) of the higher level. To ensure that the TMTFs for the higher carrier rate were obtained at the same loudness as for the lower carrier rate for each subject, the two reference current levels for the 2400 pps carrier rate stimuli were then obtained by loudness balancing with the two 600 pps reference stimuli using the following procedure.

In the loudness-balancing procedure, the 600 pps and 2400 pps unmodulated stimuli were presented in continuous alternation (with 500-ms silent periods between). The subjects adjusted the current of the 2400 pps stimulus using an up/down toggle switch until they were satisfied that the two sounds were equally loud. Subjects were encouraged to use a bracketing method (finding levels both higher and lower in loudness) on each trial before making a final decision. Next, to minimize any response bias toward the adjusted stimulus, the 2400-pps stimulus was fixed at the balanced level found on the previous run and the 600-pps stimulus was adjusted. These two runs were repeated (for a total of four runs), and the average current difference between the two stimuli over the four trials was used to set the current level for the 2400-pps reference stimulus.

#### 2.3.3 Determining modulation detection thresholds

Before the modulated stimuli were used in the modulation-detection task, each modulated stimulus of differing modulation depth was loudness balanced to the appropriate reference stimulus. The loudness-balancing procedure described above was conducted for each subject and for each combination of carrier rate, modulation frequency, overall level, and modulation depth. Modulation depths (peak to trough) used for loudness balancing and MDT measurements were initially 1, 5, 10, 30, and 50 CL (−41, −26.5, −20, −9, and −3 dB in 20log[m] units).

Next, the method of constant stimuli was used to obtain psychometric functions (one for each carrier rate, modulation frequency, and level) of modulation depth versus percent-correct modulation discrimination. The three stimuli presented on each trial were separated by 500 ms. Two intervals contained the reference unmodulated stimulus, and one randomly chosen interval contained an amplitude-modulated stimulus. Current-level jitter in the range ±4 CLs was applied in each interval. Since level jitter can detract attention from the percept of interest (modulation in this case), and modulation detection itself is dependent on level, it was important to choose a jitter range that was the minimum needed to limit loudness cues. The range chosen was based on an initial estimate of the confidence interval in the loudness-balancing task (i.e. the size in CL of the potential offset between the loudness-balanced level and the true equally loud level) and a method described by Dai and Micheyl (2010). The results section has a detailed analysis of whether the jitter range was sufficient in all cases. Subjects were instructed to identify which of the three intervals contained the different stimulus, whilst ignoring the variations in loudness. Each point on the psychometric function represented the average percent-correct identification over 25 trials for one modulation depth. For some subjects, each modulation depth was tested in a separate block of 25 trials, with modulation depths presented in a random order. For other subjects, the trials for different modulation depths were conducted in a single block of 125 trials with modulation depth pseudo-randomized (25 trials for the five different initial modulation depths).

The psychometric functions were plotted, and the MDT was defined as the modulation depth for which 70%-correct discrimination (estimated by linear interpolation) occurred. For those conditions where the 70% point occurred between 1 and 5 CLs modulation depth, additional data were collected for modulation depths of 2, 3 and 4 CLs (including loudness balancing), and the modulation depth for 70% correct was re-interpolated. When scores exceeded 70% correct for 1 CL modulation depth, the 70% point was linearly interpolated using a virtual data point of 33% correct (chance score) at zero modulation depth. The MDTs for all the conditions were tested in arbitrary order over multiple sessions. The MDTs in CLs were converted to the standard measure of modulation depth (20log[m], or dB re 100% modulation), for plotting the TMTFs.

It was not the intention to directly compare data collected with and without loudness cues. However, a subset of conditions at low and high modulation frequencies was re-measured for four of the subjects (S1, S2, S3, and S5) to assess how much results differed when limiting or not limiting loudness cues. The MDT procedure without limiting loudness cues was identical to that described above (a method of constant stimuli) with the exception that the levels of the modulated stimuli were adjusted so that the average current was the same as that for the reference stimulus, and no level jitter was used.

## 3 Results

**Fig. 1:**
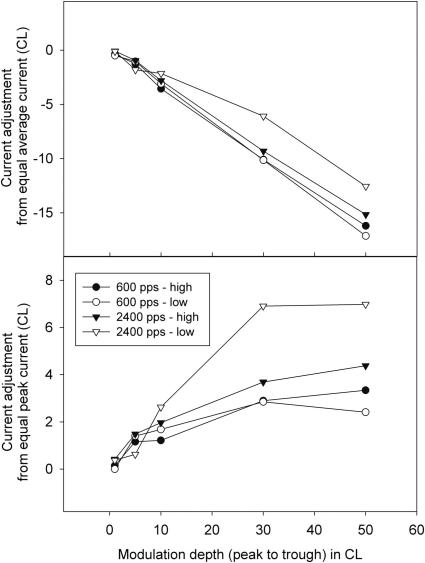
The current adjustment (in CL) of the modulated stimuli needed to equate them in loudness to the unmodulated stimulus. The top panel shows the adjustment of the modulated stimulus from a level where the average current was equal to that of the reference stimulus. The bottom panel shows the adjustment from the level for which the peak current was equal to that of the reference stimulus.

Table 2 shows the thresholds, DR and reference levels used for each subject. Fig. 1 (top panel) shows the average current level adjustment (from equal average current in reference and test stimuli) applied to the modulated stimuli to equate them in loudness with the reference. For illustrative purposes only, the data were averaged across subjects and the three modulation frequencies (50, 150, and 300 Hz) common to both carrier rates, and are shown separately in Fig. 1 for the different carrier rates and levels in the dynamic range. The data were averaged across modulation frequency as the adjustment required was similar at different modulation frequencies for each subject. It can be seen that the adjustment was highly dependent on modulation depth. The standard deviation (SD) of individual-subject means (representing how the influence of modulation on loudness differed among subjects) increased with increasing modulation depth: SDs were always less than 1 CL when the modulation depth was 1 CL, and increased to 2.0, 1.6, 2.8, and 4.8 CL for the four carrier rate/level conditions of 600/high, 600/low, 2400/high, and 2400/low, respectively at the largest modulation depth of 50 CL. The adjustment values in the top panel reflect the size of the loudness inequality that would have occurred if the modulation were applied around the mean current without adjustment of levels to equate loudness, as was done in most previous studies. The bottom panel in Fig. 1 shows the difference between the peak current levels of the modulated stimuli and the current level of the equally loud reference stimulus. Comparing the size of the adjustments in the two panels, it is apparent that the loudness of the modulated stimuli, at these carrier rates and levels, was more closely related to the peak current than to the average current in the stimuli. A two-way repeated-measures ANOVA for the 50-CL modulation depth, using individual-subject means, showed a significant effect of carrier rate (*F*(1,5) = 8.82, *p* = 0.03) but not of level, and a non-significant interaction of carrier rate and level.

The loudness-balancing data were analyzed to assess whether the jitter range (±4 CL) was sufficient in all cases to effectively limit the use of loudness cues. Dai and Micheyl (2010) analyzed the relation between jitter range (*R*), offset between test and reference stimuli along the physical stimulus dimension related to the unwanted percept (Δ), and the predicted unwanted percent correct (Pc_unwanted_) that an ideal observer would achieve when basing her selection on the unwanted percept. In the modulation-detection task here, Δ was the difference in CL between the modulated stimulus used in the detection task and the modulated stimulus that would have been exactly the same loudness as the reference stimulus. The loudness-balancing procedure ensured that Δ was as close to zero as possible (i.e. within experimental error), thus limiting the range of jitter required. Given that the loudness balance procedure is unlikely to produce ‘perfectly’ equal loudness, there will always be a residual Δ of unknown size and sign. In the analysis below, the *maximum* potential size of Δ for each modulated stimulus (at each modulated depth) was estimated using the standard errors (SEs) and derived 95% confidence intervals (±1.96∗SE) of the mean balanced levels obtained from the four repeated balancing runs. Our threshold criterion for modulation detection was 70% correct responses, therefore it was important that Pc_unwanted_ was less than 70%, and ideally less than 50% correct (chance level was 33%).

**Fig. 2:**
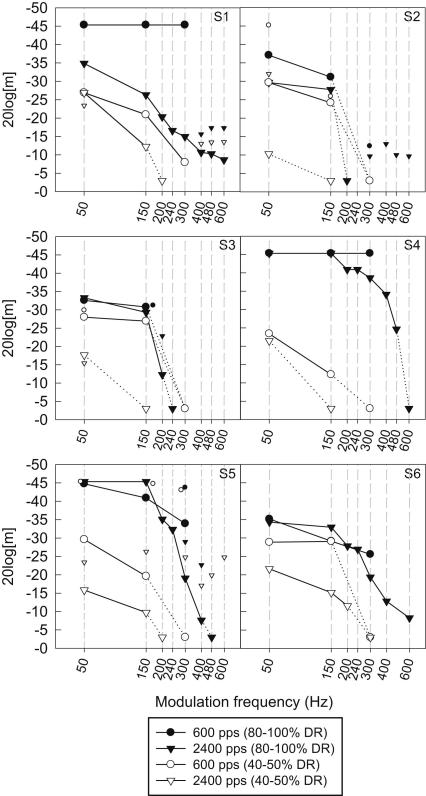
Temporal modulation transfer functions (TMTFs). Each panel shows data for one subject and contains four functions for the two carrier rates and references levels (larger symbols connected with lines). Conditions in which the largest modulation depth could not be discriminated were assigned a value of −3 dB and joined to the remainder of the function with a dotted line. The additional smaller symbols for S1, S2, S3, and S5 show selected MDTs measured *without* limiting of loudness cues. The conditions (carrier rate and level) are for the same as for the matched larger symbol type. Some small symbols are nudged horizontally to improve readability.

Under the criterion applied by Dai and Micheyl, the task used in this study was a three-interval oddity task (which of the three stimuli was the different one?) rather than a three-interval forced-choice task (e.g. which of the three stimuli was louder or higher pitch? etc). For a three-interval oddity task, the ratio Δ/*R* that leads to Pc_unwanted_ of 70% is 0.7 (that is, *R* must be greater than 1.4∗Δ to ensure that Pc_unwanted_ is less than 70%). Similarly, to ensure that Pc_unwanted_ is less than 50%, *R* must be greater than 2.3∗Δ. In our experiment *R* was 8 CL. Therefore, Δ was required to be less than 5.7 CL, and ideally less than 3.5 CL. The grand average of SEs across all subjects and conditions was 0.86 CL, with subject means ranging from 0.5 to 1.4 CL. Thus, the average 95% confidence interval for Δ was ±1.7 CL, with subject averages ranging from ±1 to ±2.7 CL. Therefore, it is highly likely that the jitter level was more than adequate for most data points. A detailed look at individual SEs (for each data point) identified one TMTF point (S5, 2400 pps carrier rate, modulation frequency of 200 Hz, 80% level) where SEs were up to 4.7 CL for some modulation depths: however, this MDT (see Fig. 2) was clearly not inconsistent with the remainder of MDTs in that TMTF. All other individual SEs were less than 2.6 CL (confidence interval less than ±5 CL).

Fig. 2 shows the individual TMTFs with the MDTs expressed as 20log[m]. Note that there were several instances where data were affected by either ceiling or floor effects, as modulation depths less than 1 CL or greater than 50 CLs were not used. In instances where 100% scores were obtained at 1 CL modulation depth, the MDT was set to −45.3 dB (equivalent to the modulation depth obtained by linear interpolation between 100% and chance score of 33% on the psychometric function, as described above). When the subject did not score above 70% correct at the maximum modulation depth tested (50 CLs), the MDT was set to −3 dB (equivalent to 50 CL). The latter ‘virtual’ data points are joined to the TMTFs with a dotted line so that the shape of the TMTF can be seen, but are not included in any analyses.

Fig. 2 also shows a subset of MDTs measured without limiting loudness cues for S1, S2, S3, and S5 (these are represented by the smaller matching symbols that are not connected by lines). At the low modulation frequency (50 Hz), two subjects (S1 and S3) showed MDTs similar to those found when limiting loudness cues. S2 and S4, however, showed a large improvement in MDT when loudness cues were not limited. At the high modulation frequencies (300 Hz and above for the 2400 pps carrier, for S1, S3, and S5), the data collected without loudness limiting show performance significantly above chance and relatively constant across frequencies from 300 to 600 Hz for each of the four cases where this was measured (S1, S2 at the high level and S1, S5 at the low level). Three of these four cases showed significantly above-chance performance at modulation frequencies more than double those that produced chance performance when limiting loudness cues. The data measured without limiting loudness cues are not included in the further analysis below.

**Fig. 3:**
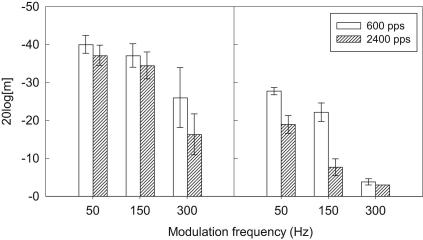
Mean MDTs expressed in dB relative to 100% modulation (20log[m]) for modulation frequencies of 50, 150, and 300 Hz. The left panel shows means for the two carrier frequencies at the higher level, and the right panel shows means for the same conditions at the lower level. Error bars show ±1 standard error of the mean. Note that the means in this figure include the points set to −3 dB: those cases where there the subject could not detect modulation at the maximum modulation depth tested, as explained in the text.

Fig. 3 shows the mean data across subjects for only those modulation frequencies (50, 150, 300 Hz) that were common to both carrier rates. The results for the two different reference levels are shown in separate panels. It should be noted that, at the lower overall level, only one of the participants (S1) was able to detect the 300-Hz modulation at the maximum modulation depth, and only at the lower carrier rate. Therefore statistical analysis of the effect of carrier rate for 300 Hz at the lower level could not be completed. However, for illustrative purposes, Fig. 3 does include the ‘virtual’ data points at 300 Hz (set to −3 dB) as these represent ‘best performance’ possible in these conditions. The possible influence of floor and ceiling effects on other statistical analyses are discussed below.

A three-way, repeated-measures ANOVA was conducted, with factors carrier pulse rate (600 and 2400 pps), carrier level (lower and higher) and modulation frequency (50 and 150 Hz). There were significant effects of modulation frequency [*F*(1,5) = 81.95, *p* < 0.001], carrier level [*F*(1,5) = 29.97, *p* = 0.003], and carrier pulse rate [*F*(1,5) = 21.43, *p* = 0.006]. There were also significant interactions between carrier level and modulation frequency [*F*(1,5) = 7.11, *p* = 0.045], and between all three factors [*F*(1,5) = 8.37, *p* = 0.034]. In Fig. 3 it can be seen that the interactions may be due to the effect of carrier level being greater at the 150-Hz than at the 50-Hz modulation frequency, and to the effect of carrier rate being greater at 150 Hz than at 50 Hz for the lower level only. There were no significant pair-wise interactions between carrier level and carrier pulse rate or between carrier pulse rate and modulation frequency.

In view of the significant interactions, analyses were conducted to investigate the effect of carrier rate and level separately for the modulation frequencies of 50 and 150 Hz, using two-way repeated-measures ANOVAs. The ANOVA for the 50-Hz data showed significant effects of carrier level [*F*(1,5) = 39.73, *p* < 0.001], and carrier rate [*F*(1,5) = 11.91, *p* = 0.018] with no significant interaction. Paired comparisons (Holm–Sidak method with family significance of 0.05) showed the effect of level to be significant for both carrier rates but the effect of carrier rate to be significant for the lower level only. The ANOVA for the 150-Hz modulation frequency showed significant effects of carrier level [*F*(1,5) = 24.02, *p* = 0.004], and carrier rate [*F*(1,5) = 18.17, *p* = 0.008]. There was a non-significant trend for interaction between carrier level and carrier rate [*F*(1,5) = 6.17, *p* = 0.056]. Paired comparisons showed the effect of level to be significant for both carrier rates but the effect of carrier rate to be significant for the lower level only. In summary, both increased carrier rate and decreased level resulted in significantly poorer MDTs. In spite of the non-significant interaction, the effect of carrier rate was not significant for either modulation frequency at the higher level.

To investigate interactions with carrier level, two-way repeated-measures ANOVAs with factors carrier rate and modulation frequency were performed for each overall level separately. For the higher level, there was no effect of carrier rate [*F*(1,5) = 1.094, *p* = 0.34], consistent with the previous pair-wise comparisons, and a significant effect of modulation frequency [*F*(1,5) = 18.17, *p* = 0.008], with no interaction between carrier rate and modulation frequency. Paired comparisons showed no effect of carrier rate for either modulation frequency, and a significant effect of modulation frequency for the 600-pps rate (*p* = 0.04) but not for the 2400-pps rate (*p* = 0.06). For the lower level, the ANOVA showed significant effects of carrier rate [*F*(1,5) = 20.75, *p* = 0.006] and modulation frequency [*F*(1,5) = 27.62, *p* = 0.003] and no significant interaction. Paired comparisons showed a significant effect of carrier rate for both modulation frequencies and a significant effect of modulation frequency for both carrier rates.

In summary, this analysis showed that, for modulation frequencies of 50 and 150 Hz, carrier rate had a significant effect on MDTs at the lower level but not at the higher level. The effect of carrier rate at the high level may have been limited by a ceiling effect (some subjects discriminated modulation at the smallest modulation depth used for each carrier rate). For these modulation frequencies, three of the subjects achieved perfect discrimination at the smallest modulation depth, with 4 data points (out of 24) at ceiling for the 600-pps rate and 4 at ceiling for the 2400-pps rate.

As mentioned above, many subjects could not detect 300 Hz modulation at the largest modulation depth tested (50 CLs or −3 dB). The effect of carrier rate at the low level could not be assessed for the 300-Hz modulation rate as there was only one data point representing non-chance performance. At the higher level, four out of six subjects had above-chance performance for both carrier rates, and it was possible to test the effect of carrier rate for this condition with a paired *t*-test. This showed a significant effect of carrier rate at 300 Hz at the higher level [*t* = 2.72, *p* = 0.024].

Overall, MDTs became poorer with increasing modulation frequency (as expected for TMTFs), and were significantly poorer at the lower level (as previously reported). When MDTs were expressed as 20log[m], the higher carrier rate produced poorer MDTs only at the lower level for 50 and 150 Hz modulation, and at the higher level for 300 Hz modulation.

**Fig. 4:**
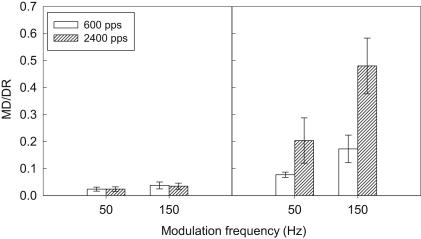
Mean MDTs expressed as a proportion of the DR for modulation frequencies of 50 and 150 Hz. The left panel shows means for the two carrier frequencies at the higher level, and the right panel shows means for the same conditions at the lower level. Error bars show ±1 standard error of the mean.

Given that DR increases with pulse rate, it was also important, for clinical relevance, to analyze the effect of carrier rate on MDTs expressed relative to the DR for each condition. This necessity arises because, in normal speech-processor use, acoustic modulation amplitudes are transformed to greater electrical modulation amplitudes when the speech processor stimulates at a higher rate. Thus, it is important to know whether the deterioration in MDTs with higher carrier rate (seen in some conditions only) is offset by the increase in DR. MDTs were therefore re-calculated as a proportion of the DR (peak to trough modulation difference in CL, divided by DR in CL). Since the maximum modulation depth (50 CLs) differed in %DR units for each subject, it was inappropriate to designate non-detection of the modulation as a single value; therefore the 300-Hz data were excluded from the analysis. Fig. 4 shows the MDTs, averaged across all participants, for all conditions except for the 300-Hz modulation frequency. A three-way repeated-measures ANOVA was performed, with factors carrier rate, carrier level and modulation frequency. There was a significant effect of modulation frequency [*F*(1,5) = 21.59, *p* = 0.006], and carrier level [*F*(1,5) = 18.74, *p* = 0.008]. There was a non-significant trend for an effect of carrier rate [*F*(1,5) = 6.32, *p* = 0.054]. Additionally, there was a significant three-way interaction [*F*(1,5) = 13.87, *p* = 0.014], and significant interactions between all paired factors: modulation frequency and carrier level [*F*(1,5) = 13.78, *p* = 0.014]; modulation frequency and carrier rate [*F*(1,5) = 11.42, *p* = 0.02]; and carrier level and carrier rate [*F*(1,5) = 6.58, *p* = 0.05].

In view of these interactions, it was necessary to investigate the effect of carrier rate separately for the four conditions in Fig. 4. Paired *t*-tests revealed that there was a significant effect of carrier rate for one condition only: 150 Hz at the lower level (*p* = 0.024). In summary, expressing MDTs relative to the DR of each subject reduced the effect of carrier rate on MDTs, so that the higher rate produced poorer MDTs only in the condition with the poorest MDTs (low level combined with high modulation frequency).

**Fig. 5:**
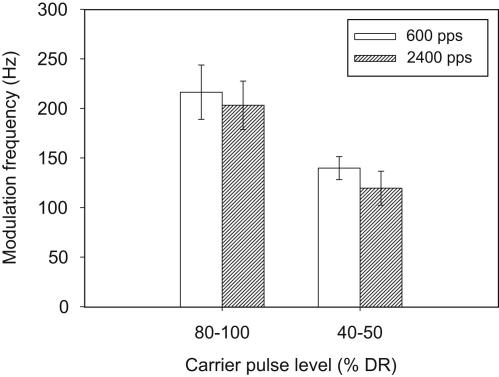
Mean cut-off frequencies of the TMTFs, defined as the modulation frequency at which the MDT was 7 dB poorer than for a modulation frequency of 50 Hz. Error bars show ±1 standard error of the mean.

TMTFs express both modulation detection efficiency (related to the absolute MDT values) and temporal resolution (related to the steepness or cut-off frequency of the TMTF). The TMTFs in Fig. 2 fall monotonically and steeply above a certain modulation frequency. To quantify temporal resolution, the steepness of each TMTF was characterized by the modulation frequency (the cut-off frequency) at which the MDTs were reduced by 7 dB from the MDT at 50 Hz. A reduction of 7 dB was arbitrarily chosen, rather than the more usual 3 dB, to produce a more robust measure allowing for the experimental error and sparse sampling of the functions. Mean cut-off frequencies across all participants for all conditions are shown in Fig. 5. Note that two subjects were able to detect 1-CL modulation at 300 Hz (the maximum frequency tested) at the high level for the 600-pps carrier rate. These particular TMTFs were assigned a cut-off frequency of 300 Hz in the analysis, representing a minimum estimate of their cut-off frequency. The effect of carrier rate and carrier level on cut-off frequency was analyzed using a two-way repeated-measures ANOVA. There was no significant effect of carrier rate [*F*(1,5) = 0.81, *p* = 0.410], and a non-significant trend for an effect of carrier level [*F*(1,5) = 6.51, *p* = 0.051]. There was no significant interaction between carrier level and carrier rate [*F*(1,5) = 0.09, *p* = 0.772]. Thus, although there was a large mean difference in cut-off frequency at high and low levels (210 Hz and 130 Hz respectively), the data were too variable to show a significant difference. The probable existence of a lower TMTF cut-off frequency for lower carrier levels was supported, however, by the significant interaction of modulation frequency and carrier level found in the previous analyses. Although there was a lower mean TMTF cut-off for higher than for lower carrier rates (161 versus 178 Hz), this difference was not significant. A trend for steeper functions at higher carrier rates was supported by the previous analysis, in which the effect of carrier rate was more evident for the higher modulation frequencies. However, more data are needed to test this possibility. It is evident from Fig. 2 that some but not all subjects showed a lower cut-off for the higher carrier rate.

## 4 Discussion

### 4.1 Modulation and loudness

The data shown in Fig. 1 demonstrate clearly that the modulated stimuli were louder than the unmodulated reference stimuli with equal average current, even for small modulation depths, and that the loudness increased with modulation depth. This demonstrates the need to limit overall loudness cues when measuring modulation detection, especially in conditions for which modulation detection would be difficult. The subset of data that was re-measured without limiting loudness cues confirms the potential for these cues to significantly improve apparent modulation detection sensitivity.

The sizes of current adjustments needed to equate loudness in Fig. 1 are broadly consistent with the data of McKay and Henshall (2010) for mid- to high-level stimuli with a carrier rate of 1000 Hz and modulation frequency of 500 Hz, and with the data of Chatterjee and Oberzut (2011) for a carrier rate of 2000 pps and modulation rates up to 200 Hz. In contrast to McKay and Henshall (2010) (and Zhang and Zeng (1997), who studied the effect using analog stimuli), there was not a large effect of overall level on current adjustment in the present results or those of Chatterjee and Oberzut (2011). This may be due to the restricted range of levels used in the latter studies. Neither the present study nor that of Chatterjee and Oberzut found a systematic effect of modulation frequency on the current adjustment to equalize loudness. The higher-carrier-rate stimuli required less adjustment to make them equally loud to the reference than did the lower-carrier-rate stimuli, consistent with the findings of McKay and Henshall (2010).

Chatterjee and Oberzut (2011) examined the effect of level jitter on MDTs for 5 subjects using an adaptive three-interval forced-choice procedure, and a carrier rate of 2000 pps. Unlike the current study, the modulated stimuli were not first loudness balanced with the reference stimulus, so that, for the poorer MDTs in that study, larger amounts of level jitter were needed to limit potential loudness cues than for the better MDTs. The authors also measured the current adjustment needed to equalize the modulated stimuli in loudness with the reference stimulus. Given the large individual variability in both MDTs and current adjustment for equal loudness evidenced by both the present study and that of Chatterjee and Oberzut, it would be difficult to calculate *a priori* the necessary range of level jitter to use in an MDT task if the stimuli were not first balanced in loudness. Using level jitter alone to limit loudness cues requires greater jitter levels than used in the current study for two reasons. The potential offset in loudness between reference and test stimuli is larger and, as mentioned above, difficult to estimate. Secondly, the offset is always positive (modulated stimulus always louder) and varies with modulation depth, in contrast to the situation after loudness balancing, where the offset is small and can be randomly positive or negative. In the latter case, the subject cannot ‘choose the louder stimulus’ when using the loudness cue, but must ‘choose the different loudness’. As Dai and Micheyl (2010) explain, the n-interval oddity task requires significantly less jitter than required for the n-interval forced-choice task to limit the unwanted loudness cue due to the same offset between test and reference stimuli.

Chatterjee and Oberzut (2011) postulated that the significant effect of jitter on MDTs in their subjects was generally due to the task being more difficult with jitter, rather than because the subjects were using loudness cues when jitter was not present. In support of this, they noted that jitter did not affect MDTs at lower levels more than at higher levels, as expected if loudness cues were more prominent for larger modulation depths. However this argument does not take into consideration the strong effect of level on intensity discrimination. Although the current offset is larger at lower levels (due to the greater modulation), the ability to *detect* the offset is restricted by the poor intensity discrimination at the lower level. Since modulation detection is strongly correlated with intensity discrimination at different levels (Galvin and Fu, 2009) it is not surprising that Chatterjee and Oberzut found that the effect of jitter on MDTs was not strongly level dependent. For the same reason Chatterjee and Oberzut's other finding cited in support of their postulation, that the subjects with poorer modulation sensitivity did not show greater use of loudness cues, is not surprising and does not therefore support their argument that loudness cues were not being used. The fact that two of the four subjects tested at low modulation rates without limiting loudness cues in the present study showed very large improvements in performance compared to when doing the task while limiting loudness cues, the latter using a jitter range less than the smallest used by Chatterjee and Oberzut, argues against the ‘cognitive only’ effect postulated by these authors.

As mentioned in the Introduction, the influence of loudness cues is predicted to become greater for higher modulation rates. Unlike the case for the effect of level, intensity discrimination is not influenced by modulation rate, so as the MDT becomes poorer, the relative salience of modulation and intensity cues changes in favor of the intensity cues. The influence of loudness cues at higher modulation frequencies is clearly seen in Fig. 2 for all four subjects who were re-tested without limiting loudness cues.

### 4.2 Effect of modulation frequency

The TMTFs shown in Fig. 2 have a common characteristic in that, apart from some data at the high level with the lower carrier rate (where 300 Hz was the maximum modulation frequency that could be tested), all show a very clear low-pass characteristic with a steep slope. Furthermore, for 17 of the 24 TMTFs, chance performance was reached at modulation frequencies ranging from 150 to 600 Hz. This result differs from those in previous reports, in which modulation at high frequencies was apparently detectable by most subjects. The TMTFs of Cazals et al. (1994) showed very little change in MDT between 200 and 800 Hz modulation. Although the influence of loudness cues in the data of Cazals et al. cannot be assessed with certainty, the similarity of the MDTs tested without limiting cues in the present experiment to those of Cazals et al., and the large difference in MDTs at these high modulation frequencies seen in the present experiment with and without loudness cues, suggest that the flat part of the TMTFs found by Cazals et al. for high modulation frequencies reflected the influence of loudness cues. Busby et al. (1993) measured TMTFs for various pulse rates and pulse durations. Only two of the seven subjects showed a consistent low-pass characteristic across conditions, indicating the possibility that subjects may have been listening to loudness cues.

Chatterjee and Oberzut (2011) did not test modulation rates above 200 Hz with jitter. Using a modulation rate of 200 Hz, 7 of the 10 MDTs were measured with sufficient jitter to limit loudness cues (using criteria described in Results section), and in 4 of these 7 cases, the jitter resulted in a steeper slope of the function between 100 and 200 Hz than obtained using no jitter. Although the authors stated that the shape of the TMTF was unaltered by level jitter, their data was limited to low modulation frequencies in 5 subjects, and the current data contradict this assertion.

The TMTFs in Fig. 2 are more clearly low-pass in shape than those for CI users tested without limiting loudness cues in most previous reports. The comparison of TMTF shapes to those for normal hearing is compromised by differences in the effects of peripheral filtering in acoustic hearing (and thus any influence of spectral cues) and differences in acoustic or internal noise effects. Viemeister (1979) showed that, for normal-hearing listeners and broadband noise carriers, MDTs for low modulation frequencies were fairly constant at approximately −25 dB and above the cut-off frequency (64 Hz, defined as 3 dB down from the plateau) increased with a constant slope of 3 dB per octave. If the criterion for cut-off frequency used in this study were applied to the functions of Viemeister (modulation frequency at which the MDT was 7 dB worse than at 50 Hz), the cut-off frequencies would be around 200 Hz, similar to the average cut-off frequency (210 Hz) found for higher levels in this study. However, it is likely that the shapes of acoustic TMTFs using broadband noise are influenced by the relationship between the modulation and the inherent fluctuations in the noise (Dau et al., 1997). In contrast, TMTFs using sinusoid carriers, which do not have inherent envelope fluctuations in the carrier, are non-monotonic, showing flat MDTs up to about 100 Hz (at least at higher levels), followed by a low-pass section with cut-off around 150 Hz and slope of 5–8 dB per octave, after which the MDTs begin to improve again because of audible spectral side-bands (Kohlrausch et al., 2000). The MDTs using electrical pulse trains are likely to be analogous to those obtained with a sinusoidal carrier, but without the influence of the spectral side-band cues. If this assumption is made, it would be predicted that the electrical functions should be flat up to around 100 Hz modulation (at high levels) and then become poorer at 5–8 dB per octave, leading to a 7-dB-down frequency of 180–200 Hz. This is broadly consistent with the average measured cut-off frequency (210 Hz) at the higher level for the cochlear implant data, although there was a wide range across subjects (from 152 Hz to more than 300 Hz). It should be noted that many of the implant TMTFs showed decrements of more than 3 dB between 50 and 150 Hz modulation frequencies, particularly at low levels, and thus resemble the TMTFs for noise carriers or for sinusoidal carriers at low acoustic levels. In summary, when comparing temporal resolution as defined by the cut-off frequency and slope of the TMTF, there is no evidence from the current data that implantees, on average, have reduced temporal resolution ability compared to normal-hearing listeners when listening at a comfortably loud level.

### 4.3 Effect of level

The level within the DR at which the data were obtained had two effects on MDTs: the MDTs were significantly poorer at the lower level, and the slope of the TMTFs was greater (as shown by the carrier level/modulation frequency interaction). These two effects imply that both modulation detection efficiency and temporal resolution are affected by the level of the stimulus. Several reports have shown a degradation in MDT with decreasing level (Chatterjee and Robert, 2001; Galvin and Fu, 2005, 2009; Pfingst et al., 2007, 2008). It is not surprising that the effect of level found in this study is broadly consistent with that found in earlier studies, since the previously-reported effect of level was very large and the greater effect of modulation on loudness at high levels compared to low levels may be counteracted, on average, by smaller modulations at threshold at high levels.

Considering the present data for 50-Hz modulation and 600-pps carrier rate, the average MDT (and standard deviation) was −40 dB (5.8 dB) at the higher level and −27.7 dB (2.4 dB), at the lower level indicating an average decrement in MDT between high and low levels of 12.3 dB. For the 2400-pps carrier rate, the average MDTs at the higher and lower levels were −37.1 dB (6.6 dB) and −19.0 dB (5.7 dB), respectively, indicating a decrement of 18.1 dB. Pfingst et al. (2007) measured MDTs for 12 implantees and at multiple electrode sites using carrier rates of 250 and 4000 Hz and a modulation frequency of 40 Hz. The average decrement when the level was decreased from 70 to 30%DR was about 10 dB for both carrier rates, which is similar to the present results for 600 pps but smaller than the decrement for 2400 pps. Pfingst et al. reported an overall greater effect of level at the higher compared to the lower carrier rate, but this effect was not evident in the region between 70 and 30%DR. In contrast, Galvin and Fu (2005) found a significant interaction between carrier rate and level for six subjects with approximate decrements in MDT from 75%DR to 35%DR of 5 dB and 15 dB for carrier rates of 250 Hz and 2000 Hz, respectively, and using a modulation frequency of 20 Hz. Galvin and Fu (2009), using a subset of the same subjects, but including additional carrier rates of 500 and 1000 pps and modulation frequencies between 2 and 100 Hz, found no interaction of the effects of modulation frequency and level. Thus the previous effect of level found in Galvin and Fu (2005) was shown to not depend on modulation frequency (for frequencies less than 100 Hz). Comparison of the current data with these published studies shows that measuring MDTs using a method that limits loudness cues did not change the general pattern of the effect of level: there was a consistent and significant degradation of MDT with decreasing level. The significantly larger effect of level at higher carrier rates seen in the current data is in agreement with Galvin and Fu (2005, 2009) but not wholly with the more extensive data of Pfingst et al. (2007). It is possible that, for both the studies of Galvin and Fu and the present study, the effect of level at the lower carrier rate and low modulation frequencies was somewhat limited by a ceiling effect in the data at the higher level. There are many differences between the studies apart from the use of a measurement method to limit loudness cues in the current study. These include the carrier rates and modulation frequencies used and the way that DR and %DR were calculated. However, it is clear that the size of the effect of level on MDTs is broadly consistent across studies.

The mechanism underlying the large effect of carrier level on modulation detection needs further investigation. Viemeister (1979) showed that TMTFs for normal-hearing listeners obtained using broadband carriers were invariant with level except at an extremely low level (spectrum level of 0 dB SPL), for which the MDTs were uniformly 3 dB poorer than those for all higher levels. In contrast, Kohlrausch et al. (2000) measured TMTFs using 10-kHz sinusoidal carriers and showed MDT decrements of 10–20 dB with decreasing level from 75 to 35 dB SPL. This decrement is of the same order as seen in the CI data if the size of the level change (75–35 dB SPL) is expressed as a proportion of DR. The usual explanations for the different effect of level with broadband and sinusoidal carriers are that, as level increases, the number of auditory filters that can be used to detect the modulation increases, and the upward spread of excitation from the sinusoid grows expansively with level leading to auditory filters in the region above the characteristic frequency of the sinusoidal carrier becoming increasingly sensitive to amplitude modulation (Kohlrausch et al., 2000). The first of these arguments could be applied in the case of electrical stimulation, as the spread of excitation grows with level and thus there are more places across the cochlea carrying the modulation information, although, since the neural excitation is already spread significantly across the cochlea, it would be expected that the effect of level would be smaller than for with acoustic stimulation. Something similar to the second factor could also apply to electrical hearing as, for low pulse rates, the slope of the loudness growth with current (on a log–log scale) becomes steeper at higher levels. However, the same argument would predict that there would be less effect of level on MDTs for high than for low carrier rates, since the slope of the loudness growth function is more constant with level for high than for low pulse rates (McKay et al., 2003). This prediction is opposite to what was seen in the data.

Studies have demonstrated a strong correlation between low-frequency MDTs and intensity difference limens (DLs), with both measures significantly worsening at lower levels (Donaldson and Viemeister, 2000; Galvin and Fu, 2009). This is not surprising as detection of a very low-frequency modulation is nearly the same as detection of a difference in two fixed levels. Signal detection theory predicts that intensity DLs would be influenced both by the growth of neural excitation with current level and by the variability in neural response. However, DR (related to loudness growth slope) is not highly correlated with intensity DLs across subjects (*r* = 0.5 found by Nelson et al., 1996), and is not consistently correlated with MDTs across electrode positions (Pfingst et al., 2008). It seems plausible, then, that changes in neural response variability with level must significantly contribute to changes in DLs and MDTs with level in addition to any contribution of changes in neural response growth with level. It is possible that lower current levels induce neural activation that is more variable over time, making it harder to discriminate changes in activation level in both DL and MDT tasks. Additionally, the lower TMTF cut-off frequencies found at the low level may be hypothesized to be related to the temporal characteristics of the increased neural response variability: the fluctuations in neural response may be of relatively high frequency, thus affecting higher more than lower frequency MDTs.

### 4.4 Effect of carrier rate

Three previous studies investigated the effect of carrier rate on MDTs. Pfingst et al. (2007) compared MDTs for carriers of 250 and 4000 pps for 12 subjects and three stimulation sites, using 40-Hz modulation: Galvin and Fu (2005) compared MDTs for carriers of 250 and 2000 pps in 6 subjects at a single cochlear site, using 20-Hz modulation: and Galvin and Fu (2009), using 5 of the 6 previous participants, compared MDTs for carriers of 250, 500, 1000, and 2000 pps, using modulation frequencies from 5 to 100 Hz. Galvin and Fu (2005) found an average decrement for the higher rate carrier of approximately 10 dB in the lower half of the DR, with slightly less decrement at higher levels (5–10 dB). There was variability in the effect of carrier rate across subjects, with one showing no effect and one experiencing an approximately 20-dB decrement. Pfingst et al. (2007) showed a mean decrement of 5.5 dB for the higher compared to the lower carrier rate, but there was considerable variability across subjects and conditions, with a range of 25.3 (lower rate better) to −12.8 (higher rate better).

Considering first our data for 50-Hz modulation, the decrement for the higher relative to the lower carrier rate was small (3 dB) and non-significant at the higher level, and larger (8.7 dB) and significant at the lower level. These decrements are of the same order of magnitude as those reported for previous studies. However, when the modulation depths were re-calculated as a proportion of the DR, there was no significant decrement for the higher compared to the lower carrier rate at either level, although there remained a non-significant mean difference in favor of the lower rate at the lower level. Therefore, there is no strong evidence from these data that perception of low-frequency temporal envelope cues conveyed by a speech processor would be harmed significantly in practice by the use of a higher carrier rate, especially since many of these cues (such as syllable offset and onset) have a large amplitude.

For the modulation frequency of 150 Hz, the effect of carrier rate on MDTs was not significant at the higher level (mean of 2.6 dB better for the low rate), but was significant at the lower level (mean of 14.4 dB better for the low rate). The advantage for low rates at the low level remained when the modulation depths were converted to proportion of DR. 300 Hz was the lowest modulation frequency to show a clear advantage (10 dB) for the low rate at the higher level. This pattern of results suggests that the perception of higher-frequency temporal cues (such as modulations at the fundamental frequency) would be better when using a lower than a higher rate of stimulation. In practice, though, this result does not lead to a way of improving signal processing, as the transmission of these high-frequency modulations requires a carrier rate at least four times the modulation frequency (McKay et al., 1994). Thus, to transmit fundamental frequency information up to 300 Hz, a carrier rate of at least 1200 pps per electrode is needed. The results do suggest, however, that the use of very high stimulation rates in a speech processor may be counterproductive. The steepness of the TMTFs measured in this study suggests that implantees are unlikely, on average, to be able to use temporal modulations above about 300 Hz, so attempting to transmit such modulations using a higher carrier rate than 1200 pps is unlikely to be fruitful.

The effect of carrier rate on transmission of temporal modulations has also been shown in an animal model using cortical physiological measures (Middlebrooks, 2008). The underlying mechanism for the degradation at high carrier rates is unclear. It has been suggested that high-rate carriers should improve the perception of temporal modulation information due to better sampling of the waveform, and also through mechanisms such as stochastic resonance (Rubinstein and Hong, 2003). The fact that higher carrier rates degrade perceptual transmission of high-frequency modulations may be due to higher-rate carriers inducing a more variable neural response (temporal envelope fluctuations), similar to the hypothesized contribution to the effect of level proposed above.

### 4.5 Temporal modulations and speech perception

Table 2 shows speech perception performance for five of the six participants. Subjects S1 and S6 had the best speech perception in quiet and S1, S6 and S3 had sentence scores that were the least affected by background noise. There was no apparent relationship between temporal modulation sensitivity at low modulation frequencies or the cut-off frequency of the TMTF and speech perception performance. There are not sufficient data here to perform a valid correlation, but the lack of relationship appears to be in contrast to the very high correlations found by Fu (2002) for nine subjects (*r* = 0.985 for consonants). It is unclear what the reason for this difference might be. Since both studies involved a small number of subjects, it is possible that the difference may be partly or wholly due to the particular choice of subjects. Since the results of Fu (2002) have yet to be replicated in the literature, there is a need to study the relation between modulation sensitivity or temporal resolution and speech understanding in a larger subject group.

## 5 Summary and conclusions

MDTs measured while limiting loudness cues showed the following characteristics:

• MDTs were similar in some instances but considerably poorer in other instances to MDTs measured without limiting loudness cues, demonstrating that the ability to detect modulation can be severely overestimated if the use of loudness cue is not limited.
• TMTFs showed very little ability of subjects, on average, to detect modulations with frequencies of 300 Hz and above. This contrasts with some previous studies, in which flat TMTFs or TMTFs with extended ability to detect modulations above 300 Hz were observed. The latter pattern was replicated in four TMTFs in the current study when re-measuring MDTs without limiting loudness cues, thus demonstrating the likelihood that the TMTFs previously measured were affected by the use of loudness cues at high modulation frequencies.
• TMTFs had shapes consistent with those obtained acoustically with wideband noise carriers or high-frequency sinusoidal carriers if spectral cues were limited for the latter. Temporal resolution, as defined by the cut-off frequency of the TMTF, was similar to that of normal-hearing listeners, at least at the higher level.
• There was a significant effect of level within the DR, as seen in earlier reports, with a large degradation in modulation detection efficiency at lower levels.
• Higher carrier rates also degraded modulation detection efficiency, with most effect at lower levels and higher modulation frequencies. When expressed as a proportion of DR, MDTs for the lowest modulation rate (50 Hz) were not significantly different for different carrier rates at either level, leading to the conclusion that using a higher carrier rate in a speech processor would not harm transmission of low-frequency envelope cues. However, the use of high carrier rates (greater than 1200 pps) may limit the perception of fundamental frequency information.

## Figures and Tables

**Table 1:** Details of subjects. The last two columns show the speech perception scores (not available for S5): percent correct phonemes in CNC words in quiet; signal-to-noise ratio (SNR) at which sentence scores were halved from the sentence scores in quiet.

Subject	Age (years)	Gender	Duration of deafness (years)	Duration of implant use (years)	Etiology	CNC words % correct	Sentences SNR (dB)
S1	64	M	7	5	Viral infection	82	7.2
S2	53	F	7	4	Familial progressive	50	11.4
S3	66	F	12	4	Idiopathic progressive	54	9.2
S4	52	F	20	5	Congenital progressive	56	10.1
S5	50	F	5	5	Idiopathic sudden	–	–
S6	76	M	5	4	CSOM	77	9.4

**Table 2:** Thresholds (T level) and dynamic ranges (DRs) of the reference stimuli for each carrier rate, and the two reference levels used for each carrier rate (low level = 40–50%DR; high level = 80–100%DR).

Dynamic range and thresholds (in CL)					Reference levels (in CL)			
Subject	600 pps DR	600 pps T level	2400 pps DR	2400 pps T level	600 pps low level	600 pps high level	2400 pps low level	2400 pps high level
S1	86	117	117	69	175	200	145	180
S2	38	152	58	126	167	182	149	172
S3	51	128	81	94	154	180	135	175
S4	68	113	89	77	145	165	122	160
S5	62	150	89	112	175	200	156	195
S6	76	106	105	68	140	186	127	165
